# Analyzing hate speech dynamics on Twitter/X: Insights from conversational data and the impact of user interaction patterns^☆^

**DOI:** 10.1016/j.heliyon.2024.e32246

**Published:** 2024-05-31

**Authors:** António Fonseca, Catarina Pontes, Sérgio Moro, Fernando Batista, Ricardo Ribeiro, Rita Guerra, Paula Carvalho, Catarina Marques, Cláudia Silva

**Affiliations:** a Instituto Universitário de Lisboa (ISCTE-IUL), ISTAR, Lisbon, Portugal; b Instituto Universitário de Lisboa (ISCTE-IUL), Lisbon, Portugal; c INESC-ID, Lisbon, Portugal; d University of Jordan, Amman, Jordan; e ISCTE-Instituto Universitário de Lisboa and Center for Psychological Research and Social Intervention (CIS-ISCTE), Lisbon, Portugal; f ISCTE-Instituto Universitário de Lisboa and Business Research Unit (BRU-ISCTE), Lisbon, Portugal; g ITI-LARSyS and IST, Lisbon, Portugal

## Abstract

This paper investigates the pervasive issue of hate speech within Twitter/X Portuguese network conversations, offering a multifaceted analysis of its characteristics. This study utilizes a mixed-method approach, combining several methodologies of network analysis (triad census and participation shifts) over the network of interaction between users. Qualitative manual content annotation was applied to the dataset to dissect different patterns of hate speech on the platform. Key findings reveal that the number of users followed by an individual and potentially reads is a relevant predictor for a user's propensity to post aggressive content. We concluded also that during a conversation thread, hate speech happens significantly more within the first 2 h of interaction. Transitivity of interactions and individual expression are considerably lower as more hate speech is prevalent in conversations. Our research confirms that hate speech is usually expressed by external individuals who intrude into conversations. Conversely, the expression of hate speech of indirect type by third parties interfering in conversations is uncommon. We also found that counter-speech discourse is strongly correlated with a type of discourse that typically avoids conflict and is not privately held.

## 1 Introduction

Hate speech on social media is a significant and complex issue that become more prevalent with the rise of digital communication platforms [1,2,3,4,5]. Hate speech refers [6,7] to a discourse that demeans, intimidates, or incites violence against individuals or groups based on attributes such as race, religion, ethnic origin, sexual orientation, disability, or gender. It is often targeted at vulnerable or minority groups [4,8], and its prevalence has led to increased research in the field, particularly in the areas of regulation, computational linguistics, and discourse analysis [8,9].

With their broad reach and user anonymity, social media platforms often become breeding grounds for hate speech. These platforms' immediacy and viral nature allow hate speech to spread quickly and widely. Twitter/X presents the user with several mechanisms for user interaction and conversation building and, consequently, hate speech propagation. *Hashtags*, which link tweets by similar topics; *Threads*, enabling users to create a sequence of connected tweets in a story-flow like dialogue; *Retweets* and *Quotations*, which allow users to retweet posts by sharing them with their followers, or quote tweets to add their opinions; and *Mentions* and *Replies* for people to engage directly in conversations, facilitate user interaction and referentiation. Each Twitter/X conversation can be unique, reflecting its global user base diverse interests and backgrounds. The platform's character limit encourages concise and creative communication, often leading to dynamic and fast-paced exchanges.

Hate speech expression in Portuguese Twitter/X is not very different from other countries in Europe [10,11]. Despite efforts across the EU to control hate speech, it has been on the rise, especially online. Our work tries to address this issue by unraveling typical characteristics of user interaction on social media associated with such crimes.

The analysis of Twitter/X conversation dynamics has revealed the importance of using dynamical computational methods to monitor and extract evolving topics and events [12]. In this type of conversation, extra-linguistic indicators such as individual initiative, group characteristics, and perceived receptivity affect user participation and their role in Twitter/X chats [13]. In Twitter/X conversations, there is a dynamic structure of @reply networks, including the shifting roles of users and their response to new triggers [14].

Social Network Analysis (SNA) is a appropriate methodology for understanding the complex fabric of social structures and relationships, offering a systematic approach to unraveling the dynamics that drive interactions within networks. When understanding hate speech on social media, not only the isolated content of each publication, but mostly its conversational context, is of crucial importance. Our research extends existing mainstream literature, which has mostly focused on the automatic detection of hate speech [9] and ignores its context [15], by categorizing and typifying the user's relative positioning inside conversations involving hate speech. Thus, we did not focus in the typical text mining tasks for automatically processing text for each message. Instead, we draw from annotators experts insights to provide meaning to its contents, and aim at understanding how such messages, characterized by experts well aware of hard to automatically detect figures of speech (e.g., irony) and other subtleties of the human natural language, propagate throughout the network. Therefore, we address this important gap recognized by existing studies.

In this work we aimed at answering the following research question.” Which patterns of behavior, associated with hate speech, can be revealed by analyzing the network of interactions in a Twitter/X conversation thread?” We used a framework called participation shifts (P-shifts) [16] and triad's census on the conversation's networks [17], to analyze interaction sequences in conversations.

P-shifts refer to the way people switch roles between speaking, listening, and being an unaddressed recipient during conversations. There are sixteen types of P-shifts, which are classified based on how the second speakers get their turn. The shifts include various possibilities, such as addressing the group, addressing a third person, or speaking after being addressed. Triad's analysis, which emphasizes the interactions within subsets of three nodes, has long been central to network science. If a network is presented as a signed graph with edges having positive or negative valence, as for example positive friendship or negative hate, a notion of ’balance’ can be applied to the network structure [18]. We build on this approach, understanding hate content, as reflecting a negative relation between any two nodes and considering the interaction during conversations, to conclude about most typical antagonisms, and flow of information patterns involving hate speech. This way, our research seeks to understand better conversation structure and discourse dynamics in which hate speech is involved, which is crucial in developing effective counter-speech initiatives. By understanding how users confront each other and counter hate speech, strategies can be designed to encourage positive engagement and reduce the spread and impact of harmful content.

This paper is organized as follows: in the next section, we perform a comprehensive literature review of recent research on the topic; in Section 3, we describe our data collection process; in Section 4 we detail the data annotation process; in Section 5 we apply a general network science perspective over the conversations networks in our dataset to describe conversation dynamics using a triad census approach; in Section 6 we then apply the concept of participation shifts [16] in order to extract patterns of user positioning along conversation interaction; finally, in Section 7, we discuss the main findings and in Section 8 we conclude with implications of the study and future work.

## 2 Literature review

The definition of hate speech is not univocal; In this paper we use the working definition developed by Ref. [19] within the kNOwHATE^1^ project: online hate speech refers to bias-motivated, derogatory language that spreads, incites, promotes, or justifies hatred, exclusion, and/or violence/aggression, targeting groups or individuals based on their group membership (e.g., perceived characteristics as ethnicity, race or sexual orientation). This definition is based on existing scholar definitions [20] and the guidelines provided by the Council of Europe in its latest Recommendation [21]. Offensive speech is also difficult to characterize. Contrary to hate speech, in offensive speech the target is not attacked because of perceived membership in a given social group, but because of a specific behavior or action [19]. Counter-speech, on the other hand, is generally associated with any direct response to hateful or harmful speech that seeks to weaken it [22].

From a computer science perspective, hate speech research has already a substantial bulk literature and has recently gained much attention [23,24,25]. A comprehensive survey of this subject can be found in recent papers such as [26]. This literature is however mostly centered on the automatic detection of hate speech using machine learning algorithms.

From a computer science perspective, research about online hate speech has been gaining interest in the last decade with the proliferation of social media. There is a significant corpus of research concerned with dataset extraction and building [27,28], and different approaches have been developed by researchers to automatically detect hateful social media content in order to build datasets and particularly for the more general purpose of automatically detect hate speech in order to tackle its impact [25]. These methods include lexicon-based, distributional semantics, multi-features, and neural networks, but the most successful methodologies involve deep learning methods and graph embedding techniques. The findings indicate that initially, the SVM algorithm and various types of TF-IDF features were the most widely used. However, after the advancement in deep-learning technology, a rapid change in the analysis methods was observed [29]. From 2017 to 2021, several comparative studies have shown the merits of deep-learning models, including CNN and RNN using word2Vec, GloVe, and FastText, among other embedding, as compared to traditional machine learning models such as SVM, LR, NB, and RF models [30]. Other approaches for multi-modal content have also been devised. The visual expression of hate, particularly *meme* detection, has also been subject to substantial research [31].

In [32], the authors explore how conversations among individuals in a social network can reveal important details about the network's structure. How groups form, the activity within these groups, individual roles, and the likelihood of someone joining a conversation are all things analyzed in this study. Using Twitter/X as an example, the study uses the timing of messages to connect participants in the network, introducing a new way to visualize how well the community is coordinated or synchronized based on participants' timing and social connections. This paper highlights the methods and insights used to contribute to the growing field of Dynamic Social Network Analysis.

The work presented in this paper follows this perspective of research. Prior research about online social media dialogue, conversations, and interaction, not all related to hate, from 2010 onwards, has covered various dimensions of Twitter/X threads, ranging from conversation structural attributes [33,34,35,36] to user's influence, opportunity, authority and power [37,38,39]. The existing body of work around Twitter/X conversations related to hate encompasses a diverse range of topics covering the prediction of user participation in conversations [40], the relationship between structure and toxicity in conversations [41]; the prediction of the amount of hateful propagation [42,43,44,45]; counter hate speech analysis [46] or the characterization the trajectories of individual discussions on Facebook [47]. Our study is more related to this last paper and is located within the domain of Twitter/X interaction. We focus on user positioning on exchanging hate speech as an aspect that, while it has been explored to some degree, still needs further investigation due to its evolving nature and relevance.

## 3 Data collection

Although nationality was the most frequently cited attribute mentioned in discrimination complaints, several reports show that race/nationality, sexual orientation, and gender identity were the strongest motivations for hate crimes in Portugal [48,49]. Our dataset was built focusing on four target groups of hate speech: *racialized* communities in general, and the *Roma* community in particular, *migrants* and *LGBTI +* communities. These groups were defined due to their relevance in Portuguese social media hate speech discourse [10,11] and cited in the major reports about this topic [1,2,3].

The collection of Twitter/X data was performed using the Twitter/X API with a research/academic license. To perform this extraction, we compiled a list of 259 keywords associated with the target groups to retrieve tweets containing these keywords. This list was obtained from a previous research project about hate speech [50]. To select potential targets, we considered first only the unambiguous words corresponding to 174 entries in the keyword list. Ambiguous words were not selected in this first retrieval since they can have different meanings depending on the context. We then associated ambiguous words with insults from a predefined list of common and uncommon insults with approximately 800 entries. Data collection was limited to a two-year span from January 1, 2021, to December 31, 2022. The language was filtered to Portuguese, predominantly resulting in Portuguese from Brazil (pt-br) instead of Portuguese from Portugal (pt-pt). To ensure geographical relevance, and limiting the context to the Portuguese case, the collection was narrowed to tweets posted in Portugal.

Additionally, tweets belonging to the same conversation of the previously retried tweets were also retrieved, focusing only on those for which the seed tweet was published in Portugal. The conversation tweets were also collected using the Twitter/X API with its specific filter. Our final dataset consists of complete conversations with a parent tweet published in Portugal, resulting in a total of 29531 tweets.

## 4 Data annotation

All the above tweets were annotated relying on annotation guidelines developed specifically for the purpose of identifying key social psychological and linguistic features of online hate speech and counter speech [50]. This annotation was performed by an interdisciplinary team of researchers with backgrounds in linguistic and social psychological. These annotators meticulously identified different linguistic elements as for example: the hate speech; if indirect or direct, offensive speech, and several others rhetorical and emotional features. Reliability coefficient Krippendorff's alpha (*α*) was used to evaluate the level of agreement between annotators. These values are presented in Table 1. The agreement levels for all features varied from moderate to low. These alpha values may not solely be due to the difficulty of classifying subjective data but could also be attributed to the lack of enough data variability [51]. Binary variables, where one value is significantly rare (in our study, the presence of a phenomenon, coded as 1, has a low frequency), exhibit low variability. Consequently, even if there is agreement in the annotation, the alpha coefficient tends to be low. Additionally, the annotators identified the target community mentioned in messages, shedding light on the intended recipient or subject. Each tweet can have more than one type of speech, depending on the context and content.

**Table 1:** Krippendorff's alpha values for the five different types of speech annotated.

Type of speech	*α*
Hate Speech	0.355
Direct Hate Speech	0.195
Indirect Hate Speech	0.211
Counter Speech	0.501
Offensive Speech	0.163

**Fig. 1:**
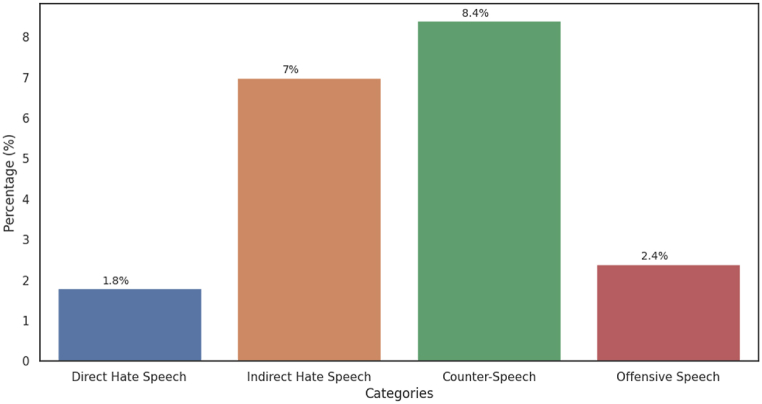
Distribution of the type of discourse identified. Offensive speech, Direct Hate speech, Indirect Hate speech or Counter hate Speech.

After the annotation, we did a preliminary analysis that showed the prevalence of tweets with no toxic or toxic-related speech, representing almost 83 % of the dataset. These tweets we included in the conversations because they belonged to the threads, but in fact they did not have any hate content. Regarding the distribution of speech types in the dataset, Direct Hate Speech and Offensive Speech have a small representation. However, the values rise when looking into Indirect Hate Speech and Counter Speech, as we can see in Fig. 1.

## 5 Conversation dynamics

**Fig. 2:**
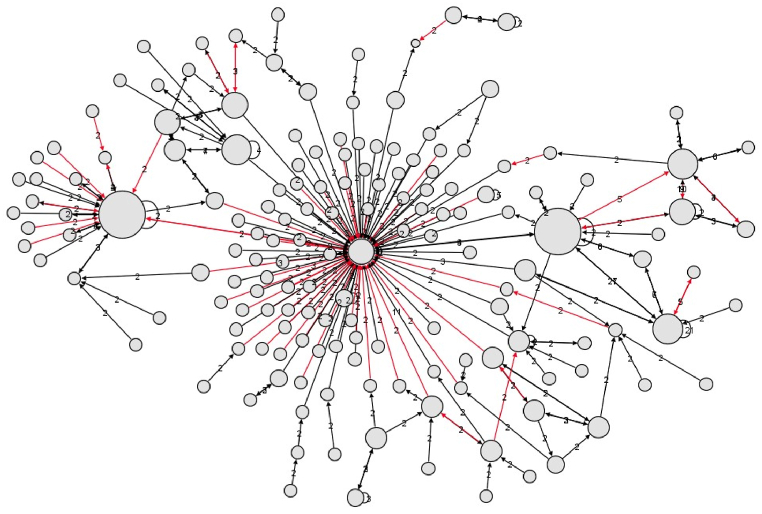
Example of a conversation that contains 233 tweets. This conversation was extracted from the Twitter/X API using tweets with the same conversation ID number. Each node corresponds to a user, and each edge corresponds to a tweet reply between users. In red tweets with some form of offensive or hate speech. The size of the nodes is proportional to the out degree (number of replies).

Based on the collected dataset, we build conversation networks similar to the one in Fig. 2. We made sure to leave out any conversations with fewer than three tweets since they do not constitute a good representation of a conversation. This left us with a dataset containing 1967 conversations and 28323 tweets. Each conversation contains a certain number of hate speech tweets classified by the annotators and a certain number of users. We performed a logistic regression taking as dependent variable the number of tweets annotated with hate speech content and independent variables: (a) the *Presence* of each user on the social network, calculated as the number of posted tweets divided by the number of days since the account was created; (b) the number of *Followers*, roughly quantifying the user's popularity and (c) the number of followed accounts by the users. All these quantities were collected form the user's public Twitter/X profiles. The results are listed in Table 2.

**Table 2:** Logistic regression for the posting of hate tweets given each user presence on Twitter/X, the number of followers and the number of users he/she is following.

Characteristic	log(OR)^2^	95 % CI^2^	p-value
Presence	2.2	0.06, 4.3	0.041
Followers	−0.54	−2.9, 1.1	0.6
Following	4.4	2.8, 5.9	<0.001

From these results, we conclude that there is no significant influence of user popularity or user publication presence/activity on Twitter/X on the production of hate speech. On the other hand, the number of users each user follows, and potentially also reads, is a strong predictor of the user's propensity to post aggressive content. This may constitute an important finding already reported by other studies [52,45].

**Fig. 3:**
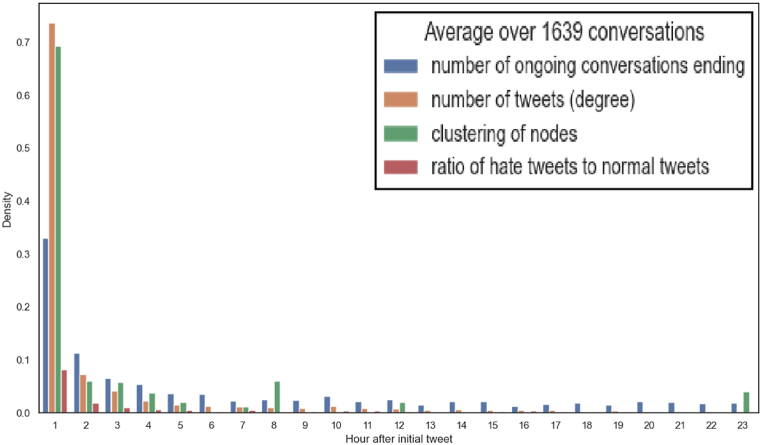
Hourly frequency of active conversations for 1639 conversations not longer than one day. Each mark represent an additional hour over the time the first tweet in the conversation was posted.

We evaluated some network metrics on a subset of our original dataset constituted by the set of conversations that lasted less than one day. This subset represents 83 % of the original dataset and presents some interesting characteristics that are depicted in Fig. 3. The first time series is associated with the density of activity in these conversations during the following 24 h after an initial tweet is posted. Examining the chart, we can see that although many conversations extend past the first hour, most of the activity occurs during that hour, after which it decays very quickly. We measured not only the number of conversations ending but also the number of tweets produced in the hour (average out-degree) and the average clustering coefficient within the network of tweets at each hour. This last measure quantifies the density of the conversation sub-network, within the whole conversation network, which is active for each hour. We can see that it also decays but does not necessarily follow the number of tweets posted. Concerning the expression of hate, we can also verify that it happens almost exclusively in the first couple of hours. However, as this type of speech represents a small proportion (13.5 %) of the overall conversation, it is difficult to conclude any other characteristic.

**Fig. 4:**
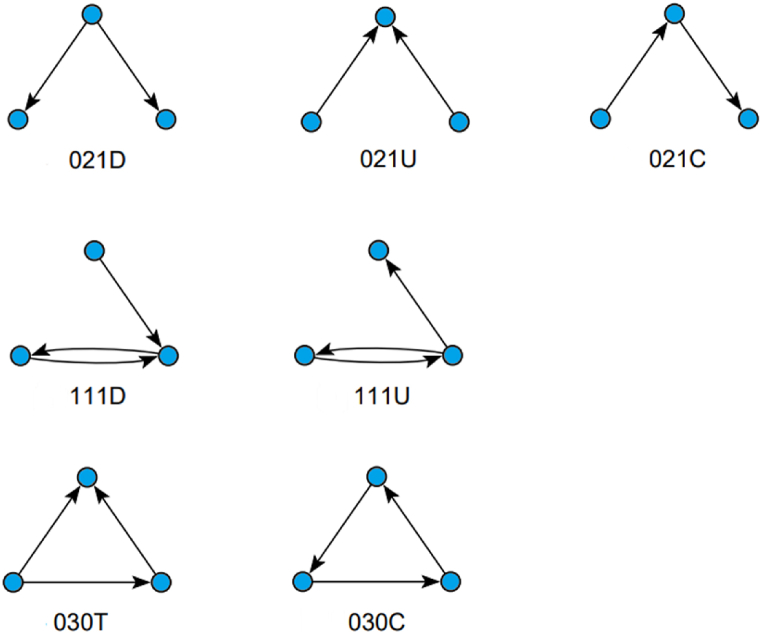
Triad coding.

We examined further a triad census for each of the 1964 conversation networks, aiming to find particular motifs of tweet interaction. Table 3 lists a comparative percentage between similar types of triads for three different conversation networks progressively more complete: conversations networks not including counter speech or hate speech tweets, conversations networks not including counter speech tweets, and the complete conversations networks. Possible combinations between three types of triads associated with two and three tweets are compared (see Fig. 4: Triad coding). More trivial or more complex possible triads are not assessed as their interpretation would be more ambiguous.

**Table 3:** Census of triads concerning two or three tweets and conversations networks without hate and counter-hate speech tweet edge, conversations without counter-hate speech tweet edge, and complete conversations.

Triad	Conversations without hate speech and counter speech	Conversations without counter speech	Complete conversations
021D	0.39 %	0.22 %	0.17 %
021U	98.22 %	98.75 %	98.99 %
021C	1.39 %	1.03 %	0.83 %
111D	93.11 %	94.06 %	95.57 %
111U	6.89 %	5.94 %	4.43 %
030T	89.08 %	92.92 %	91.43 %
030C	10.92 %	7.08 %	8.57 %

In the first case concerning two tweet triads (021D, 021U, and 021C), the structure of interactions is quite obvious. We can see that individual expression archetypal triad (021D) in which an user replies to others, or information exchange archetypal (021C), in which users interact transitively, lowers as the conversation network includes more hate speech related content.

In the second case concerning three tweets and a dyadic exchange between a pair of users (triads 111D and 111U), we can see that there is more interference to the user's exchange (111D) and less individual expression (111U) as the conversation network includes more hate speech related content.

In the third case (triads 030T and 030C), transitivity and information exchange (030C) lowers as the conversation network includes more hate speech related content, confirming the first case.

## 6 User positioning - P-shift classification

Participation shifts (P-Shifts) [16] constitute categories of user positioning in a conversational context based on how the second speaker takes their turn after the first speaker speaks to someone. First, a person may speak after being directly addressed (turn-receive). Second, a person may speak after someone else is addressed, assuming the target of a remark is expected to speak next (turn usurping). Third, a person may speak after someone addresses the entire group (turn claiming). Lastly, when someone already speaking changes their focus, it is called turn continuing (See Table 4). It is important to note that although P-shifts are often discussed regarding two speaking turns, turn-continuing P-shifts happen within a single turn.

**Table 4:** Listing of the different types of participation shifts (P-Shift) according to Gibson 2003 [16].

P-shift^3^	
Turn receiving	
AB-BA	John talks to Mary, then Mary replies.
AB-BB	John talks to Mary, then Mary talks to herself.
AB-B0	John talks to Mary, then Mary addresses the group.
AB-BY	John talks to Mary, then Mary talks to Irene.
Turn claiming	
A0-X0	John talks to the group, then Frank talks to the group.
A0-XA	John talks to the group, then Frank talks to John.
A0-XY	John talks to the group, then Frank talks to Mary.
Turn usurping	
AB-X0	John talks to Mary, then Frank talks to the group.
AB-XA	John talks to Mary, then Frank talks to John.
AB-XB	John talks to Mary, then Frank addresses Mary.
AB-XY	John talks to Mary, then Frank addresses Irene.
Turn continuing	
A0-AY	John talks to the group, then addresses Mary.
A0-AA	John talks to the group, then talks more.
AB-A0	John talks to Mary, then makes a remark to the group.
AB-AA	John talks to Mary, then talks again.
AB-AY	John talks to Mary then to Irene.

Twitter/X data fits this type of analysis because all the modes of participation shift listed can be easily extracted from the different modalities (reply, mention, comment, and retweet) of user interaction. We performed a p-shift census over the conversation dataset which supported a statistical analysis using logistic regression.

### 6.1 Relation between conversation positioning and type of discourse

**Fig. 5:**
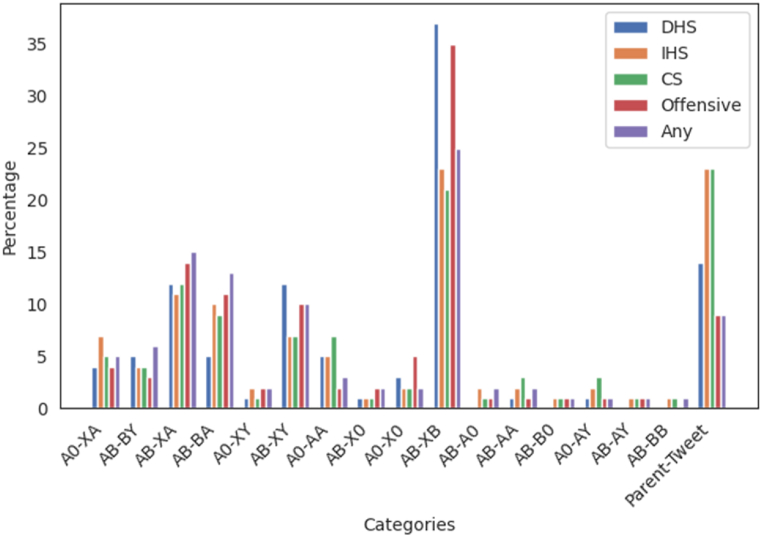
Distribution of the type of discourse by P-shift. Offensive speech, Direct Hate speech, Indirect Hate speech, Counter Speech and any other type.

Our Twitter/X data (See Fig. 5) indicates that *Offensive Speech* and *Hate Speech* tweets are more likely to fall into the AB-XB category, showing that offensive discourse tends to be originated by an outside party to conversations and targeted at the addressed speaker, confirming other authors [41]. The greater than average proportion on p-shift AB-XY also confirms this finding.

Another interesting finding is that *Indirect Hate Speech* and *Counter Speech* are more frequent than the average in cases of seed tweets of conversations or reiterated addressing to all (A0-AA). An interesting finding is that indirect hate speech is much more frequent in initial seed tweets than in the directed type.

*Counter Speech* is also more prevalent when addressing a third person after speaking to all (A0-AY) or reinforcing the addressing to someone (AB-AA) or to all (A0-AA).

The graph gives a detailed comprehension of how speech patterns dynamically transition throughout the conversation, offering essential insights into the evolution of communication styles.

After this census, we performed a logistic regression modeling of *Direct Hate Speech* discourse against the P-Shift mode in order to find statistically significant associations (Table 5). The analysis confirms that *Direct Hate Speech* is mainly expressed by external individuals in the conversation. Interestingly, results also show that *Direct Hate Speech* may occur outside the context of the conversation between two external speakers (second row of Table 5).

**Table 5:** Logistic regression model of Direct Hate Speech Discourse. The independent variables are the P-shift modes of the tweets, the dependent variable is Direct Hate Speech presence on tweets.

P-shift	log(OR)^4^	95 % CI^4^	p-value
AB-XB	0.89	0.61, 1.2	*<*0.001
AB-XY	0.93	0.57, 1.3	*<*0.001

Table 6 shows the results of the logistic regression model of *Indirect Hate Speech* discourse against the participation shift modes. Interestingly, in this case, this type of hate speech, contrary to the previous case, tends not to be expressed by a third individual external to the conversation. We do not have evidence of the opposite case, but the prevalent unfavorable odds ratio suggests that the expression of indirect hate speech by a third person is very uncommon. Also, we see that this type of discourse is negatively correlated with one type of turn claiming (A0-X0) and three types of turn usurping (AB-XA, AB-XB, and AB-XY), possibly reinforcing this idea that this type of discourse involves a certain amount of personal shared meaning. *Indirect Hate Speech* is more subtle, and existing literature recognizes that it is better understood within the context of dyadic dialogue.

**Table 6:** Logistic regression model of Indirect Hate Speech Discourse. The independent variables are the P-shift modes of the tweets, the dependent variable is Indirect Hate Speech presence on tweets.

P-shift	log(OR)	95 % CI	p-value
A0-X0	−0.69	−1.1, −0.30	*<*0.001
AB-XA	−0.39	−0.59, −0.18	*<*0.001
AB-XB	−0.42	−0.59, −0.25	*<*0.001
AB-XY	−0.47	−0.72, −0.23	*<*0.001

Table 7 shows the results of a logistic regression model of *Offensive Speech* discourse against the participation shift modes. It is possible to observe that offensive speech invariably correlates with two not-so-peaceful forms of turn shifting in conversations. Specifically, the claiming of turn by an external party (A0-X0 and A0-XY) and all forms of turn usurping (AB-X0, AB-XA, AB-XB, and AB-XY), two forms of turn receiving are also contemplated (AB-BA and AB-BY), still with lesser expression.

**Table 7:** Logistic regression model of Offensive Speech Discourse. The independent variables are the P-shift modes of the tweets, the dependent variable is Offensive Speech presence on tweets.

P-shift	log(OR)	95 % CI	p-value
A0-X0	1.7	1.2, 2.2	*<*0.001
A0-XY	1.3	0.60, 1.9	*<*0.001
AB-AY	1.4	0.44, 2.2	0.002
AB-BA	1.1	0.76, 1.5	*<*0.001
AB-BY	0.85	0.33, 1.3	*<*0.001
AB-X0	1.3	0.59, 1.9	*<*0.001
AB-XA	1.1	0.76, 1.4	*<*0.001
AB-XB	1.4	1.1, 1.7	*<*0.001
AB-XY	1.3	0.97, 1.7	*<*0.001

Finally, we tested the *Counter Speech* discourse against the different participation shift types with logistic regression, as shown in Table 8. The results show that it strongly correlates with a kind of discourse in which the speaker sends a message to all and then addresses a particular subject (A0-AY) in a turn continuation mode. On the other hand, three forms of turn usurping (AB-XA, AB-XB, and AB-XY) and two forms of turn claiming (A0-X0 and A0XA) are negatively correlated with counter speech. This type of discourse possibly avoids conflict. Significantly, the direct form of private dialogue (AB-BA) is negatively correlated with the presence of *Counter Speech*, possibly meaning that this type of discourse is not privately held, typically considering a general audience.

**Table 8:** Logistic regression model of Counter Discourse. The independent variables are the P-shift modes of the tweets, the dependent variable is Counter Speech presence on tweets.

P-shift	log(OR)	95 % CI	p-value
A0-AY	1.1	0.58, 1.6	*<*0.001
A0-X0	−0.84	−1.2, −0.45	*<*0.001
A0-XA	−0.50	−0.77, −0.23	*<*0.001
AB-BA	−0.44	−0.66, −0.22	*<*0.001
AB-XA	−0.36	−0.56, −0.16	*<*0.001
AB-XB	−0.68	−0.85, −0.51	*<*0.001
AB-XY	−0.56	−0.80, −0.32	*<*0.001

## 7 Discussion

In this paper, we extensively analyzed a conversation dataset containing 29531 messages extracted from the social platform Twitter/X. The dataset was annotated, addressing three forms of harmful speech: *Offensive Speech*, *Direct Hate Speech*, *Indirect Hate Speech*, and one form of *Counter Speech*.

The main findings we have obtained are listed as following:

1. There is no significant influence of user popularity or user publication activity on the production of hate speech. On the other hand, the number of users each user follows and potentially reads, is a relevant predictor of a user's propensity to post aggressive content. This results confirms findings in the literature [53,54], and may be explained by user's exposure to certain types of content; for example, if a user reads more aggressive posts or follows more users without being followed, he is more likely to post also aggressive content. This finding can also be explained by the prevalence of bots which normally follow many other users without reciprocity.
2. During a conversation thread, hate speech happens significantly more within the first 2 h of interaction. This also happens for the number of posts and dialogue exchange, and it does not necessarily depends on the conversation duration. This finding can be explained by the very nature of the platform, which appeals to short replies and diversity of interactions [55,41].
3. Concerning triadic analysis, we noticed that collective behavior, as individual reply to many peers and transitive interactions among three parties, is lesser when the conversations network includes more hate speech and offensive content. This can lead us to conclude that in the presence of hate speech conversation growth should be reduced. Saveski et al. [41], on the other hand, found that ”At the group level, … toxic conversations tend to have larger, wider, and deeper reply trees, but sparser follow graphs”, meaning that toxic conversations tend to expand, although its participants do not necessarily follow each other. We observed, in fact, the prevalence of third party interference on dyadic interactions in the presence of hate speech content. This may in part explain conversation build up through replies between users that, in other case, would not interact.
4. Other positioning assessment of speakers when hate speech is involved, with the concept of Participation Shifts, also reveals that direct hate speech is particularly expressed by external individuals that interfere or come from the outside of the conversation that is taking place. On the other hand, the expression of indirect hate speech by a third person is very uncommon. This can be explained by the fact that indirect hate speech may involve a certain amount of shared meaning. Offensive speech invariably correlates with two not-peaceful forms of turn positioning shifting in conversations: turn usurping and turn claiming. Finally, Counter-speech discourse strongly correlates with a kind of speech in which the speaker sends a message to all and then addresses a particular subject in an exemplary manner. We also found that this type of discourse avoids conflict and is not privately held, possibly meaning that it preferably considers a general audience.

## 8 Conclusion

This work has comprehensively explored the complexities and dynamics of hate speech on Twitter/X. Through our analysis, we have highlighted how Twitter/X, as a microblogging platform, is a double-edged sword: it facilitates free expression and information dissemination and acts as a breeding ground for harmful and hate-fueled rhetoric. Our findings emphasize the multifaceted nature of hate speech. Tackling hate speech on Twitter/X requires a multipronged approach. This includes the development of more advanced detection algorithms, in which automatic hate speech detection is concerned, implementation of stricter policies by Twitter/X, increased public awareness and education, and collaboration with legal authorities and NGOs. It is crucial for all stakeholders, including users, platform developers, policymakers, and civil society, to work together to create a safer and more inclusive online environment. Future approaches should include a more thoroughly Twitter/X user characterization other than the one present in Table 2. User detailed profiling and group participation can better help understanding hate speech phenomena.

## Data availability

The authors do not have permission to share data.

## CRediT authorship contribution statement

**António Fonseca:** Writing – original draft, Methodology, Formal analysis, Conceptualization. **Catarina Pontes:** Writing – review & editing, Writing – original draft, Investigation, Data curation, Conceptualization. **Sérgio Moro:** Writing – review & editing, Writing – original draft, Investigation, Conceptualization. **Fernando Batista:** Writing – review & editing, Validation, Data curation. **Ricardo Ribeiro:** Writing – review & editing, Validation, Data curation. **Rita Guerra:** Validation, Project administration, Data curation. **Paula Carvalho:** Validation, Data curation. **Catarina Marques:** Validation, Data curation. **Cláudia Silva:** Validation, Data curation.

## Declaration of competing interest

The authors declare that they have no known competing financial interests or personal relationships that could have appeared to influence the work reported in this paper.

## Footnotes

2 OR = Odds Ratio, CI = Confidence Interval.

3 The initial speaker is always labeled A, and the initial target B, unless the group is addressed (or the target was ambiguous), in which case the target is O. Then the shift is summarized in the form [speaker.] [target.] -[speaker.] [target.], with A or B appearing after the hyphen only if the initial speaker or target serves in one of these two positions after the shift. When the speaker after the shift is someone other than A or B, X is used, and when the target after the shift is someone other than A, B, or the group, Y is used.

4 OR = Odds Ratio, CI = Confidence Interval.

## Footnotes

1 https://knowhate.eu/.
